# Myopic Posterior Persistent Fetal Vasculature: A Rare Presentation

**DOI:** 10.7759/cureus.58623

**Published:** 2024-04-20

**Authors:** Ankur K Shrivastava, Yamini Patial, Barkha Singh, Narendra Kuber Bodhey

**Affiliations:** 1 Ophthalmology, All India Institute of Medical Sciences, Raipur, Raipur, IND; 2 Radiodiagnosis, All India Institute of Medical Sciences, Raipur, Raipur, IND

**Keywords:** leukocoria, microphthalmia, myopia, anisometropic amblyopia, persistent fetal vasculature

## Abstract

Persistent fetal vasculature (PFV) is a rare ocular developmental disorder resulting from incomplete apoptosis of the embryonic hyaloid vasculature during the in-utero period. Variability in the development and regression of hyaloid vasculature is responsible for the wide range of clinical presentation of the disorder. PFV may manifest as anterior segment abnormalities (cataract, glaucoma, microphthalmia, elongated ciliary process with central traction, retrolental membrane, and shallow anterior chamber), posterior segment abnormalities (vitreous stalk, preretinal membranes, optic hypoplasia, and retinal folds), or with a combined anteroposterior disease. The most common associated clinical feature is leukocoria with microphthalmia and usually unilateral presentation. Most of the cases have poor visual prognosis and present early in childhood. Association with myopia is a very rare and atypical presentation, especially unilateral cases which may present later in life and have a good visual prognosis. Hereby, we present a case of a 27-year-old young adult male with unilateral atypical myopic posterior PFV with anisometropic amblyopia and good functional vision in the right eye.

## Introduction

Persistent hyperplastic primary vitreous (PHPV), persistent fetal vasculature (PFV), persistent fetal vasculature syndrome (PFVS), and benign mimic of retinoblastoma all are synonymous terms for the same rare ocular developmental disorder. This ambiguity in terminology was resolved in 1997 by Goldberg by giving the accurate and clinically useful term persistent fetal vasculature, as it results from incomplete apoptosis of both the embryonic hyaloid vessels (primary vitreous) and the tunica vasculosa lentis which may induce the growth of retrolenticular fibrovascular mass of variable size hampering the proper development of eye (both anterior and/or posterior segment) [1]. It can be unilateral in up to 90% of cases and more commonly has a poor visual prognosis in the affected eye because of associated complications and progressive anisometropia [2]. Based on anatomical involvement, there are three types: purely anterior (24%), purely posterior (12%), and combined (60%). PFV has a wide spectrum of presentation and may manifest as anterior segment abnormalities (cataract, glaucoma, microphthalmia, elongated ciliary process with central traction, retrolental membrane, and shallow anterior chamber), posterior segment abnormalities (vitreous stalk, pre-retinal membranes, optic hypoplasia, and retinal folds), or with combined anteroposterior disease [3,4].

PFV remains an important cause of visual disability in children. It presents mostly in the infantile period making it the second most common cause of childhood blindness. Typically, the most common presentation is a combined form along with leukocoria and microphthalmia in childhood. The other forms are also most commonly associated with microphthalmia. In PFV, the progression of the disease does not occur after birth but the growth of the eye with age leads to various complications later on such as angle-closure glaucoma, vitreous hemorrhage, and tractional retinal detachment.

In this case report we present a rare association of purely posterior PFV with myopia with anisometropic amblyopia. Such cases are usually not associated with vision-threatening complications like in cases with leukocoria and microphthalmia [5], and therefore present later in life [6]. Visual prognosis in such cases is relatively good. These patients are screened later in life beyond visual maturation and therefore present with anisometropic amblyopia. Long-term complications, such as secondary glaucoma and intraocular hemorrhages are usually absent in such cases [5].

## Case presentation

**Figure 1:**
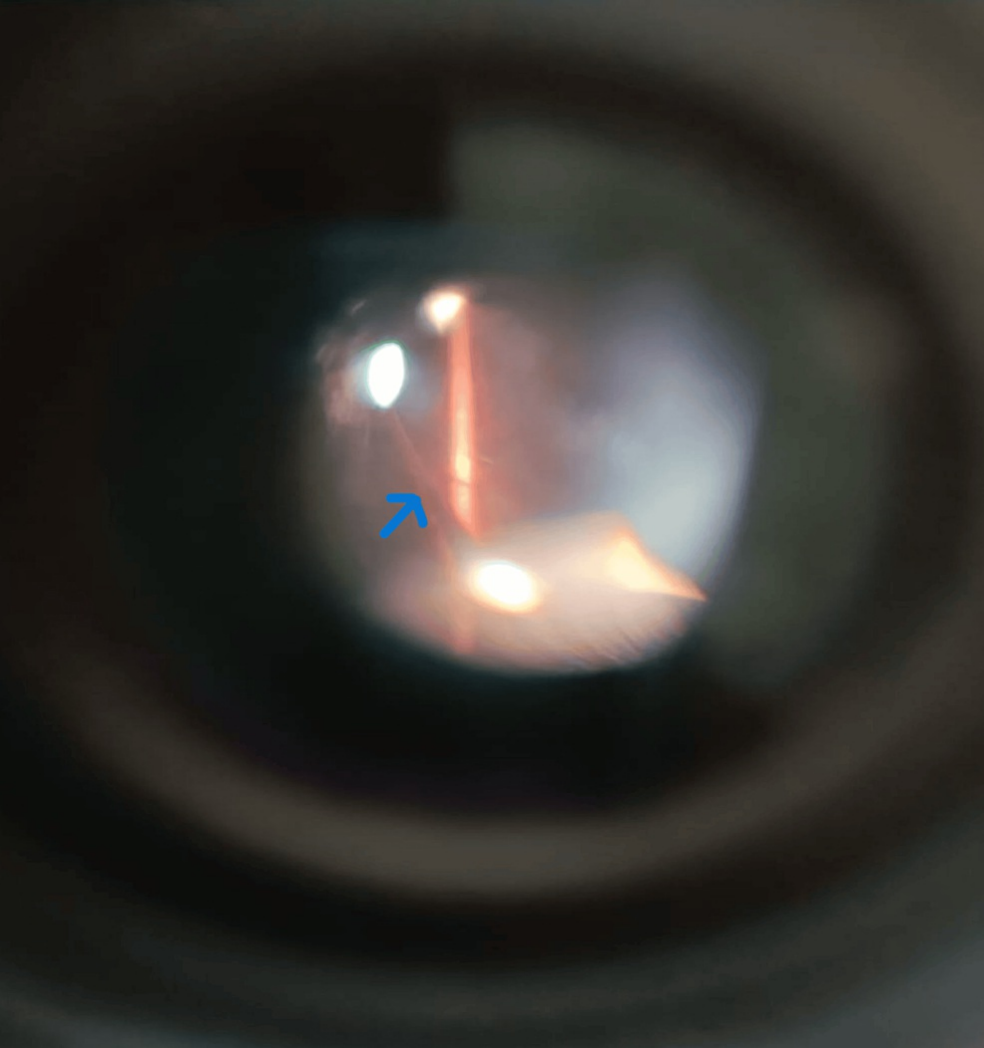
Vitreous stalk as seen on 90 D Slit lamp biomicroscopy (blue arrow).

A 27-year-old male presented to ophthalmology OPD with the chief complaint of diminution of vision in the right eye for the past 1 year, with a history of irregular use of spectacle for 6 months. The general examination was normal. Snellen’s visual acuity in the right eye was 6/24 (partial) improving to 6/18 with pinhole, and 6/6 in the left eye. On slit lamp (SL120, Carl Zeiss Meditec AG, Germany) examination, the anterior segment was within normal limits in both eyes. Intraocular pressure measurement with a Goldmann applanation tonometer was 14 and 15 mm Hg in the right and left eye respectively. Dilated evaluation of the posterior segment using a 90 D slit lamp biomicroscope showed the presence of a stalk-like structure running from the optic disc to the anterior vitreous phase in the right eye (Figure 1). 

**Figure 2:**
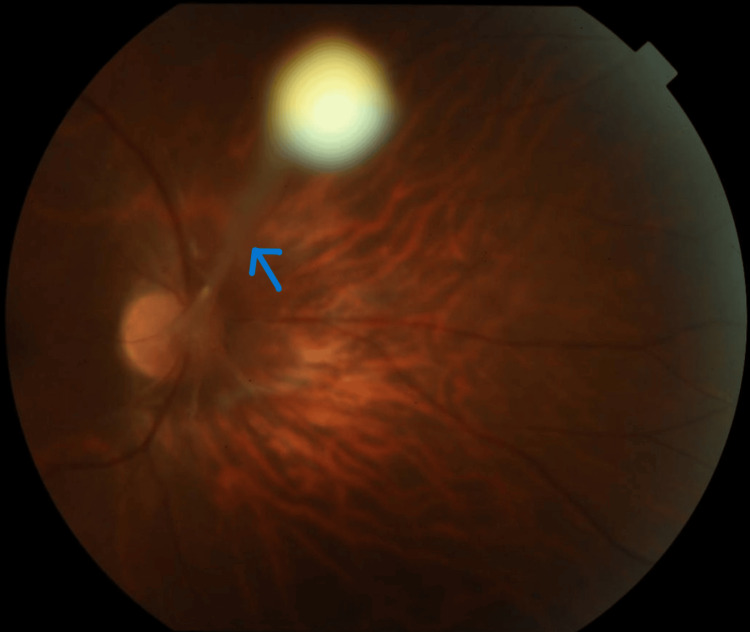
Colour fundus photograph of the right eye showing the vitreous stalk running from the optic disc to the anterior vitreous phase(blue arrow).

During an indirect ophthalmoscopy, vitreous degeneration was seen, and the rest of the peripheral retina was within the normal limits. After cycloplegic refraction, vision in the right eye improved with -1.50 DS/-0.75 DCyl x 175 till 6/18. Unaided vision in the left eye was 6/6. A fundus photo of the right eye taken using a fundus camera (Topcon retinal camera-50DX, type1A, Tokyo, Japan) revealed a stalk-like structure running from the optic disc to the anterior vitreous phase (Figure 2). 

**Figure 3:**
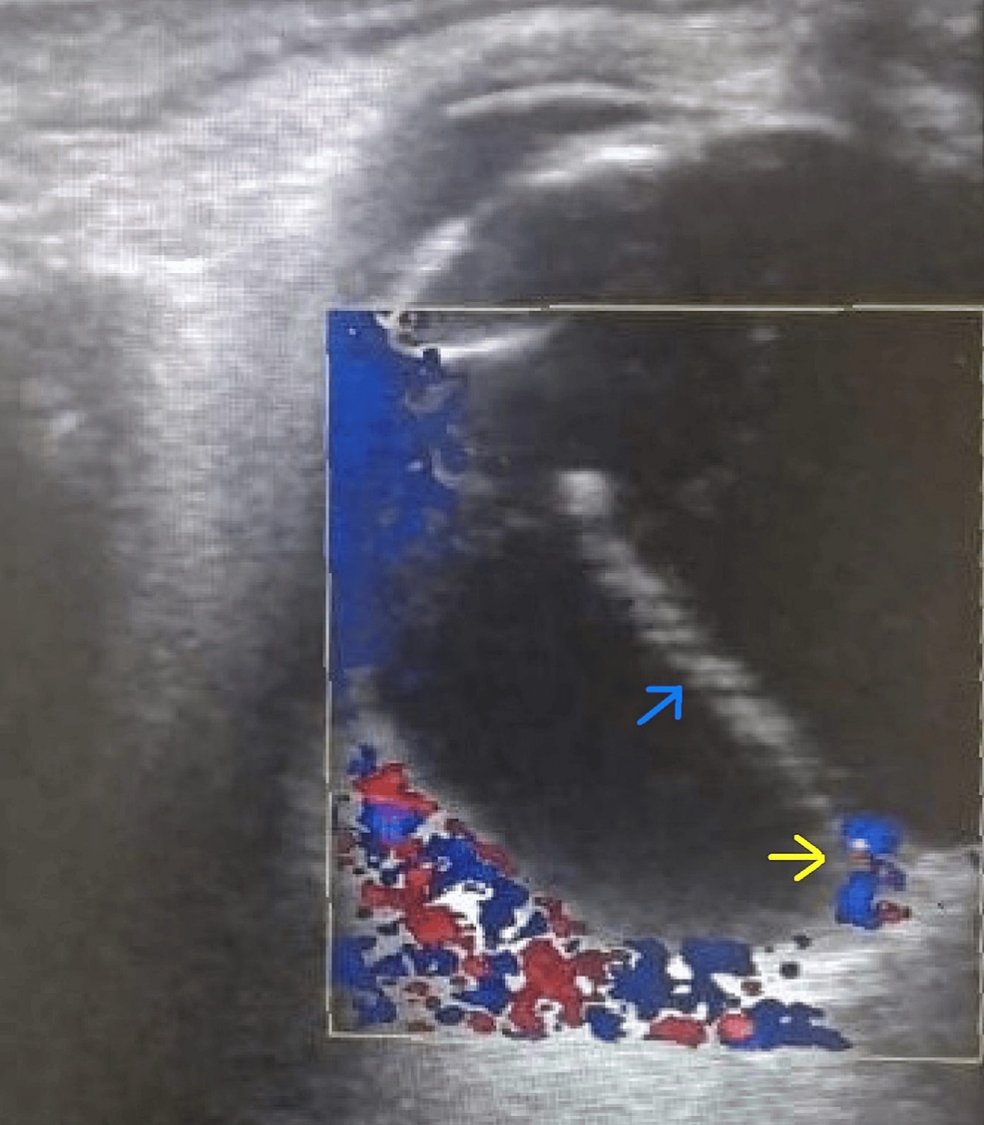
Ultrasound colour doppler of right orbit demonstrating vascular stalk running from optic disc to anterior vitreous phase(blue arrow) with corresponding internal vascularity in posterior third segment(yellow arrow) representing persistent fetal vasculature.

Partial coherence interferometry (IOLMaster500, version 5.4, Carl Zeiss Meditec AG, Germany) revealed an axial length of 24.35 mm and 23.60 mm in the right and left eye respectively, and anterior chamber depth was 3.72 mm and 3.77 mm in the right and left eye respectively. Ultrasound (Logiq S8, GE Healthcare, Sangdaewood-dong, Korea) of the right orbit revealed a linear thick echogenic band-like structure with a linear central hypoechoic area in the posterior segment of the right eye with attachment posteriorly in the optic disc and anteriorly in the anterior vitreous phase. On colour Doppler, corresponding internal vascularity in its posterior one-third segment representing persistent fetal vasculature was seen (Figure 3). 

**Figure 4:**
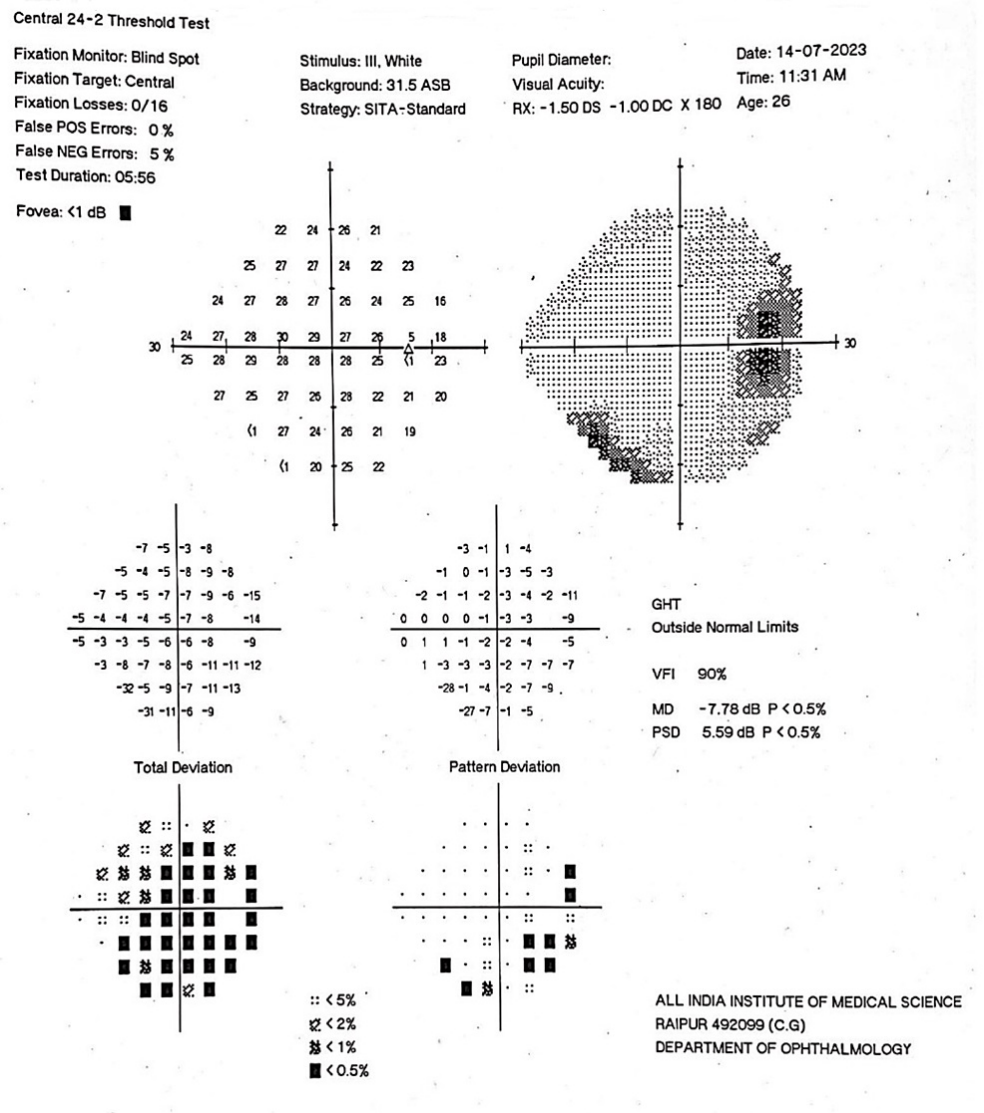
Visual field of the right eye showing no glaucomatous field changes.

This was associated with multiple floating thin echogenic bands suggestive of vitreous degeneration. Ultrasound of the left orbit was within normal limits. The visual field analysis done by Humphrey’s perimeter (Humphrey field analyzer, Model 750i, Carl Zeiss Meditec AG, Germany) was within normal limits (Figure 4).

**Figure 5:**
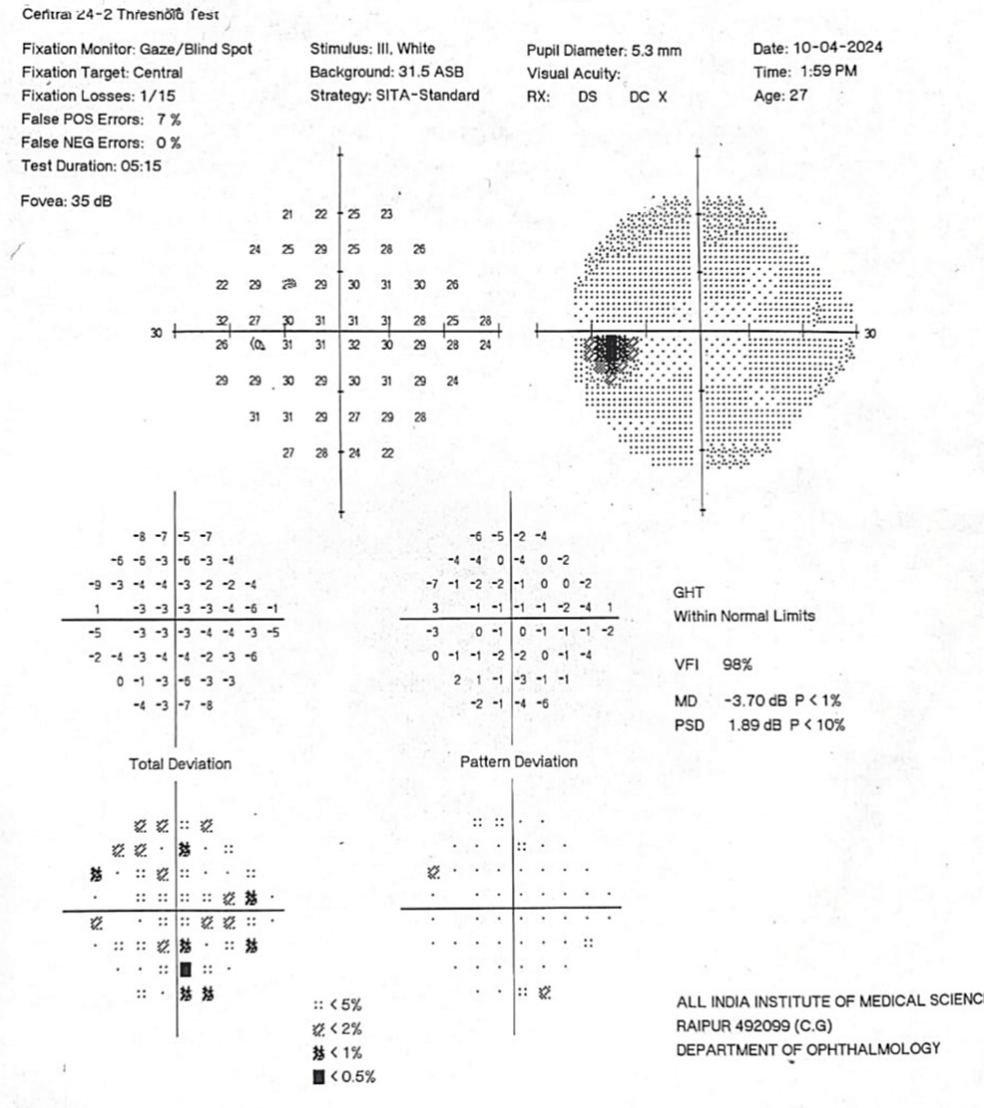
Normal Visual field of left eye

The visual field was also within normal limits in the left eye (Figure 5).

The patient is kept on regular follow-up for early detection of complications, if any, in the future.

## Discussion

Persistent fetal vasculature is a well-described disease in literature but its prevalence is still unknown as it is a rare disease. The few studies that had reported the prevalence of PFV show a large variation from 0.064% to 5% [7,8]. It is not only a rare disease but also has a diverse and broad spectrum of presentation, which usually becomes apparent at birth or shortly thereafter. Our patient was a previously healthy 27-year-old adult, which is an unusual and rare presentation of PFV, and cases that were reported in the literature with late presentation are at a much younger age than our case.

Most cases are sporadic and unilateral in presentation. Based on the site of involvement, the most common type is the combined form (involving the anterior and posterior segment); the purely posterior form is present in about 12% of cases [9]. More than two-thirds of cases of the purely posterior form are associated with microphthalmia and reduced corneal size [10]. Our patient has a purely posterior presentation with normal corneal size and without any associated microphthalmia, which is a rare association.

Another unconventional presentation is the long axial length, this myopic type is considered a rare variant [5]. In children with PFV, long axial length may present in the anterior or combined form due to long ciliary processes, or due to the development of buphthalmos secondary to glaucoma. Our patient is an adult who has a long axial length in the right eye, i.e. 24.35 mm, while the unaffected eye has a normal axial length and normal intraocular pressure in both eyes. This is not only a rare association but also contributes to amblyopia in this patient due to anisometropia. The fundus observation constituted only an isolated stalk-like fibrovascular band extending from the posterior pole with vitreous degenerative changes and a normal retina. Based on previous reports, the purely posterior form is the rarest anatomical form in PFV and has a poor visual prognosis. Predictors of poor visual prognosis in PFV are bilateral involvement, microphthalmia, glaucoma, and posterior pole involvement. However, our case shows the right eye in a purely posterior form with moderate anisometropic amblyopia, despite the patient's affected eye having good functional vision.

This case is incidentally detected during a detailed eye check-up. Heterogenicity of PFV presentation makes diagnosis challenging if a detailed eye examination is not done. Hence, all suspected patients should undergo a detailed ocular examination to diagnose such varied presentations. These patients should be kept under regular follow-up and monitored for complication development. Appropriate refractive error correction should be given and, if required, amblyopia correction therapy should be considered in children and adults [11]. Surgical management should be reserved for cases developing complications.

## Conclusions

Atypical unilateral myopic posterior PFV variants have different clinical presentations, lines of management, and prognoses as compared to the typical microphthalmic variants. In children, due to the plasticity of visual development, even small differences in axial length can lead to amblyopia. To conclude, a purely posterior form of PFV can present with myopia and stay undetected in childhood. Such cases should be screened properly and kept under observation for the development of any complications. As amblyopia is one of the treatable causes, emphasis should be given to the screening of children during the visual developmental age of suspected cases where visual acuity does not correspond to anterior segment findings, so that early detection and treatment can help in avoidance of lifelong sequelae. Surgical management should be reserved for cases developing complications and therefore, rarely required.
